# Effects of exogenous glycine betaine on growth and development of tomato seedlings under cold stress

**DOI:** 10.3389/fpls.2024.1332583

**Published:** 2024-03-22

**Authors:** Taoyu Dai, Songtao Ban, Liyuan Han, Linyi Li, Yingying Zhang, Yuechen Zhang, Weimin Zhu

**Affiliations:** ^1^ Shanghai Key Laboratory of Protected Horticulture Technology, The Protected Horticulture Institute, Shanghai Academy of Agricultural Sciences, Shanghai, China; ^2^ Key Laboratory of Intelligent Agricultural Technology (Yangtze River Delta), Ministry of Agriculture and Rural Affairs, Agricultural Information Institute of Science and Technology, Shanghai Academy of Agricultural Sciences, Shanghai, China; ^3^ State Key Laboratory of North China Crop Improvement and Regulation/Key Laboratory of Crop Growth Regulation of Hebei Province/College of Agronomy, Hebei Agricultural University, Baoding, Hebei, China

**Keywords:** cold stress, tomato seedlings, glycine betaine, hyperspectral phenotyping, cryoprotectant

## Abstract

Low temperature is a type of abiotic stress affecting the tomato (*Solanum lycopersicum*) growth. Understanding the mechanisms and utilization of exogenous substances underlying plant tolerance to cold stress would lay the foundation for improving temperature resilience in this important crop. Our study is aiming to investigate the effect of exogenous glycine betaine (GB) on tomato seedlings to increase tolerance to low temperatures. By treating tomato seedlings with exogenous GB under low temperature stress, we found that 30 mmol/L exogenous GB can significantly improve the cold tolerance of tomato seedlings. Exogenous GB can influence the enzyme activity of antioxidant defense system and ROS levels in tomato leaves. The seedlings with GB treatment presented higher Fv/Fm value and photochemical activity under cold stress compared with the control. Moreover, analysis of high-throughput plant phenotyping of tomato seedlings also supported that exogenous GB can protect the photosynthetic system of tomato seedlings under cold stress. In addition, we proved that exogenous GB significantly increased the content of endogenous abscisic acid (ABA) and decreased endogenous gibberellin (GA) levels, which protected tomatoes from low temperatures. Meanwhile, transcriptional analysis showed that GB regulated the expression of genes involved in antioxidant capacity, calcium signaling, photosynthesis activity, energy metabolism-related and low temperature pathway-related genes in tomato plants. In conclusion, our findings indicated that exogenous GB, as a cryoprotectant, can enhance plant tolerance to low temperature by improving the antioxidant system, photosynthetic system, hormone signaling, and cold response pathway and so on.

## Introduction

Cold stress is a significant environmental factor that has a detrimental impact on plant growth and productivity (Liu et al., 2012; Ré et al., 2017). When exposed to low temperature, plants undergo physiological and biochemical responses, including the production of reactive oxygen species (ROS), inhibiting photosynthesis and changes in osmotic solutes (Lu et al., 2020; Rahman et al., 2023; Xu et al., 2023). In order to survive under cold stress, plant activates cold response signals and transduction to regulatory networks which initiates multiple responses, including physiological and biochemical responses (Ding et al., 2020). These responses include hormone metabolism and signal transduction, synthesis of various protective compounds (e.g., proline and soluble sugars), enhancement of antioxidant capacity, changes in stabilization of membrane systems, and improvement of cold tolerance (Zhang et al., 2022).

Using multiple exogenous cryoprotectants to enhance plant’s tolerance to cold stress is an effective way (Román-Figueroa et al., 2021). The latest research indicates that the regulation mechanisms of cryoprotectants in plants to cold stress are complex processes, including efficiently scavenging ROS, the production of osmotic agents, activation of cold regulating (COR) genes, and so on (Jahed et al., 2023). Exogenous melatonin application improves the cold tolerance of strawberry seedlings by stimulating the expression of downstream genes in the DREB/CBF-COR pathway (Hayat et al., 2022). Moreover, the application of exogenous hormone has a positive effect (Larkindale and Huang, 2005). Abscisic acid (ABA) can increase tolerance to drought and cold stress by decreasing water loss and activating downstream signaling (Heidari et al., 2021a). Brassinosteroids(BRs) can improve the plant heat tolerance in the regulation of ROS metabolism through the expression of many antioxidant genes that enhance the activity of antioxidant enzymes (Ogweno et al., 2008).Additionally, plant secondary metabolites have been found to reduce damage from abiotic stress, such as proline, soluble sugars and so on (Kumar et al., 2011). Therefore, the study of improving plant cold resistance with exogenous substances has important practical production application value.

Glycine betaine (GB) is one of the well-known stress protectants (Park et al., 2006; Yao et al., 2018; Shemi et al., 2021). The accumulation of GB produced by exogenous or transgenic applications can induce the expression of certain stress-responsive genes, including those for enzymes that scavenge reactive oxygen species (Chen and Murata, 2011). *CodA* gene, which encodes a choline oxidase to catalyze the conversion of choline to GB, was transferred into tomato that normally does not accumulate GB. These transgenic plants had accumulated GB and are more tolerant of chilling stress than their wild-type counterparts (Park et al., 2004).Previous studies have revealed that application of exogenous GB enhances the ability to combat abiotic stresses in crops (Khalid et al., 2022). Exogenous-applied GB can improve drought tolerance in wheat during reproductive growth stages (Shemi et al., 2021). Transcriptome analysis showed it improved the plant growth through up-regulating osmoprotection, increasing net photosynthetic rate and the catalase activity, decreasing ion leakage and protecting the antioxidant defense system (Khalid et al., 2022). In tomato, exogenous GB increases the seed germination by reducing ROS formation, altering the contents of metabolites and plant hormones (Zhang et al., 2022).

Tomato is amongst the most widely cultivated vegetables, and it is highly sensitive to chilling stress (Liu et al., 2020a). In this research, we applied exogenous GB to the seedlings to evaluate the impact of GB on the cold stress during the tomato seedling stage. We evaluated the following parameters: high-throughput plant phenotyping, chlorophyll-related parameters and hormone levels, as well as the gene expression related to low temperature response. This study aims to uncover the regulatory mechanism of exogenous GB on the chilling tolerance of tomato seedlings and identified exogenous substances that regulate plant cold tolerance-related genes in order to understand their expression patterns.

## Materials and methods

### Plants and growth conditions

The material NRP20 used in this experiment is a cold-sensitive species obtained from Shanghai Academy of Agricultural Sciences. Tomato seedlings were grown in greenhouse in controlled conditions (18-h light/6-h dark cycles, 25°Cday/18°C night. And 60% relative humidity) and were treated using exogenous GB (containing 0.01% Tween-20 surfactant) one time at the four-leaf stage in order to do different experiments. Different concentrations of GB, 20 mmol/L, 30 mmol/L, 40 mmol/L, 50 mmol/L were sprayed one time on tomato seedling groups while the control was sprayed with distilled water, each group with 72 seedlings. The plants grew in the normal temperature in the greenhouse for 24 hours after GB treatment and then were subjected to cold stress (4°C) for observing the phenotype and were selected the most suitable concentration of GB. Based on the previous study, the 30 mmol/L GB solution was selected to pretreat tomato seedlings while the control group was pretreated with distilled water. Under the same culturing conditions as the mentioned above, the seedlings grew at room temperature for 24 hours before undergoing low-temperature treatment for the following experiment. They were used for measuring plant hyperspectral phenotyping, enzyme activities, chlorophyll fluorescence, hormone levels, and the gene expression.

### Morphological observation and measurement of tomato seedlings

On the 7th day, the growth status of tomato seedlings from different treatments was observed, and the survival rate and embryonic root length were measured using a vernier caliper (72 independent biological replicates were measured).

### Determination of antioxidant enzyme activities and DAB chemical staining

Leaves (5th from cotyledon) under GB and water treatment were harvested at different time points according to experimental requirements. The levels of proline content, MDA content,H_2_O_2_ content,O_2_^·-^ content superoxide dismutase (SOD), catalase (CAT), and peroxidase (POD) activities were measured using an enzyme-linked immunosorbent assay (ELISA). Each measurement was performed in triplicate, and the detection methods were performed according to the instructions from Suzhou Kemi Biotechnology Co., Ltd. Tomato seedlings at the same developmental stage were selected for 3-diaminobenzidine (DAB) staining. The 5th leaves were immediately placed in a DAB solution (pH 3.8, 1mg/ml) and subjected to vacuum infiltration until the leaves sank to the bottom. The stained leaves were then incubated at 28°C for 6-10h, during which a dark red precipitate was observed. The stained leaves were subsequently subjected to a series of washes in 95% ethanol, followed by boiling water for 10 minutes, and then subjected to three cycles of washing in 85%, 70%, and 50% ethanol solutions. Finally, the stained leaves were photographed using a camera.

### Plant hyperspectral phenotyping

The tomato seedlings was placed on a black light-absorbing background cloth in a darkroom, and a hyperspectral imaging system was used to obtain hyperspectral images of the tomato seedlings. In the environment for visualizing images (ENVI) software, the decision tree classification method was used to separate each tomato seedling area from the background in the hyperspectral image, and the chemical properties of the plant were quantitatively measured at the individual plant level. The average spectral reflectance data of all pixel reflectance in the pure tomato seedling image area were calculated as its spectral reflectance data. The partial least squares discriminant analysis (PLS-DA) was used to analyze the spectral reflectance differences of WR and GR, WL and GL tomato seedlings before and after processing. PLS-DA was used to establish a relationship model between spectral parameters and sample categories to achieve sample category prediction and establish a reliable mathematical model to summarize and generalize the spectral characteristics of the research object. The parameters of the PLS-DA evaluation model are explanatory rate of the model for the X matrices (R^2^X), explanatory rate of the model for the Y matrices (R^2^Y), and prediction ability of the model (Q^2^). The spectral reflectance of each tomato seedling was used to calculate normalized difference vegetation index (NDVI), structure insensitive pigment index (SIPI), carotenoid reflectance index 1 (CRI1), carotenoid reflectance index 2 (CRI2), pigment specific normalized difference a (PSNDa) and pigment specific normalized difference b (PSNDb), which were closely related to the plant physiology (Blackburn, 1998; Gitelson et al., 2002; Vásquez et al., 2018; Rasheed et al., 2020).

### Measurement of chlorophyll fluorescence and P700 parameters

The chlorophyll fluorescence imaging system (WALZ, IMAG-MAX/L) was used to observe and analyze the photosynthetic system of tomato leaves at the same location, measure the chlorophyll fluorescence and P700 oxidation-reduction state of the leaves. Before measurement, tomato seedlings were dark-adapted for 15 minutes. The maximum photochemical efficiency of photosystem II (PSII) after dark adaptation (Fv/Fm), the non-photochemical quenching coefficient (NPQ), the apparent photosynthetic electron transport rate (ETR) of PSII, and the photochemical quenching coefficient (qP) were determined by measuring the fluorescence intensity emitted by the PSII antenna during light energy conversion, obtaining information on the operation of PSII. Six leaves at the same location of each treatment were randomly selected, and three points were randomly selected for each leaf, avoiding the veins.

### Extraction and determination of plant hormones ABA and GA

The contents of ABA and GA of the samples at different time (0, 24, 48, and 72h) after chilling treatment were determined to use an enzyme-linked immunosorbent assay (ELISA) supplied by China Agricultural University (Zhang et al., 2022). Six independent biological replicates were tested.

### RNA extraction and real-time quantitative PCR

Total RNA was extracted from tomato seedling leaves at different time points (0, 1, 2, and 12h) after chilling treatment by the Biospin Plant Total RNA Extraction Kit (Hangzhou Bioer Technology Co., Ltd.). The RNA extraction process followed our laboratory’s experimental protocol (Zhang et al., 2022). The extracted RNA was reverse transcribed by the HiScript II One Step RT-PCR Kit (Novogene Corporation) to obtain cDNA. The *EIF* gene was used as an internal reference gene, and qRT-PCR analysis was performed using the Hieff UNICON^®^ Universal Blue qPCR SYBR Green Master Mix (Shanghai Yisheng Biological Technology Co., Ltd.). Each reaction was performed in triplicate, and the relative expression levels were calculated by 2^-△△CT^ method. The primer design was conducted using Primer5 software (see ST1).

### Statistical analysis

Data were processed with Excel, and statistical analysis was performed with SPSS 22.0 (IBM Corp., Armonk, NY, USA) with a significant level of *P* < 0.05 (Zhang et al., 2022). GraphPad Prism 8 (GraphPad Software, Inc., USA) was used for data visualization. Phenotypic observations were recorded using a Canon camera.

## Results

### Exogenous application of GB promotes the cold stress tolerance of tomato seedlings

**Figure 1:**
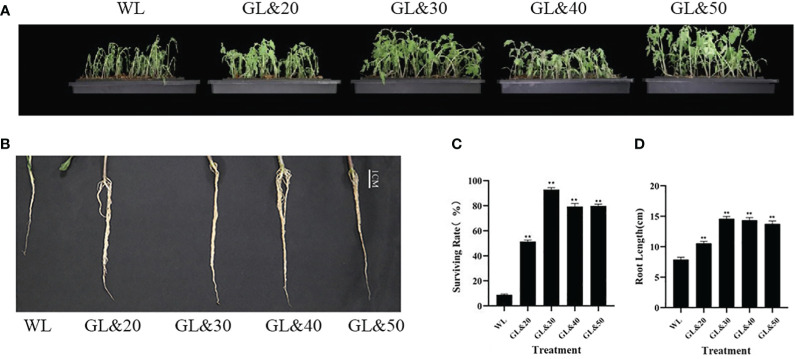
Effects of different concentrations of GB on growth of tomato seedlings under cold stress. **(A)** Tomato seedling phenotype. **(B)** Seedling root length and growth at day 7. **(C)** Survival rate of seedlings at day 7. **(D)** Seedling root length at day 7. In all cases, the asterisk indicated a significant difference among the groups according to Tukey’s test. (**P < 0.01). WL: Tomato seedlings treated with water at low temperature; GL&20: 20mmol/L GB was applied externally at low temperature; GL&30: 30mmol/L GB was applied externally at low temperature. GL&40: 40mmol/L GB was applied externally at low temperature; GL&50: 50mmol/L GB was applied externally at low temperature.

Tomato plants were subjected to water treatment and GB treatment and placed them at 4°C for 7 days. We then performed a phenotypic analysis of the overall growth status and leaf wilting degree of the tomato seedlings. As shown in **Figure 1A**, after 7 days of cold treatment, the leaves of the tomato plants treated with water had withered, while the plants treated with GB appeared good growth compared to the water-treated plants (**Figure 1A**). We subsequently measured the root length of the tomato plants with a vernier caliper and calculated their survival rates. The results demonstrated that after cold treatment, the root diameter of the tomato plants treated with water became thinner, and the root length was significantly shorter. In contrast, the survival rates of the tomato plants treated with GB (20mmol/L, 30mmol/L, 40mmol/L, 50mmol/L) were all significantly increased, while the roots of the seedlings were all longer than control (*P* < 0.01) (**Figures 1C, D**). The treatment effect of the 30mmol/L concentration was superior to that of the other concentration (**Figure 1B**). This suggested that GB treatment exerted a stimulating influence on the development of tomato seedlings under cold stress. Notably, the seedlings showed the best survival rate under the treatment of GB 30mmol/L. Exogenous GB effectively mitigates the detrimental effects of cold stress on the growth and development of tomato seedlings, fostering the growth of both the aerial and subterranean parts of the seedlings, thereby enhancing their morphological architecture.

### GB treatment enhances ROS scavenging capacity of tomato seedlings under cold stress

**Figure 2:**
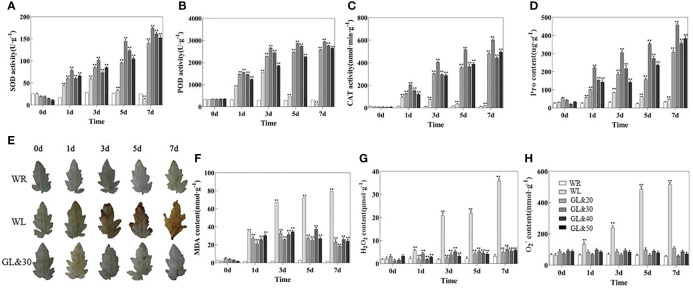
Different concentrations of GB on the accumulation of reactive oxygen species (ROS) and the activities of antioxidant enzymes in tomato seedlings at 0, 1, 3, 5 and 7 days of cold treatment **(A)** SOD activity. **(B)** POD activity. **(C)** CAT activity. **(D)** proline (Pro) content. **(E)** DAB staining. **(F)** the accumulation of malondialdehyde (MDA). **(G)** H_2_O_2_ content. **(H)**O_2_^-^ content. In all cases, the asterisk indicated a significant among the groups according to Tukey’s test. (**P < 0.01). WR: tomato seedlings treated with water at room temperature; WL: Tomato seedlings treated with water at low temperature; GL&20: 20mmol/L GB was applied externally at low temperature; GL&30: 30mmol/L GB was applied externally at low temperature. GL&40: 40mmol/L GB was applied externally at low temperature; GL&50: 50mmol/L GB was applied externally at low temperature.

When plants suffer cold stress, they induce excessive production of ROS. Leaf phenotypes were observed using DAB histochemical staining and the contents of H_2_O_2_ and O_2_^-^ were measured. The contents of malondialdehyde (MDA) and proline were also measured and compared as indicators of oxidative damage. As shown in **Figures 2F–H**, there was no obvious difference in the contents of H_2_O_2_, O_2_^-^, MDA, and proline in tomato plants at room temperature. Cold stress increased excessive production of ROS. in both treatment groups. However, we found that tomato leaves treated with GB had less accumulation of MDA, H_2_O_2_, and O_2_^-^, while a huge increase in proline content with significant differences at the rate of increase (*P* < 0.01). In contrast, the WL treatment reached maximum levels of H_2_O_2_ and MDA accumulation on the 7th day of cold stress, with a daily decrease in proline content, which reached its lowest value on the 7th day (**Figures 2D–H**). It indicates that tomato seedlings treated with GB can effectively reduce oxidative stress damage caused by cold stress. Plants initiate protective enzyme systems to scavenge excess ROS and prevent cell damage caused by abiotic stress. At room temperature, no significant differences were discovered among SOD, CAT and POD activities in tomato seedlings. However, the activities of antioxidant enzymes in tomato seedlings were significantly enhanced under cold stress, and the activities of SOD, POD, and CAT were observed to be higher (*P* < 0.01) than in the GB-treated group compared to the WL treatment group (**Figures 2A–C**). It indicates that under cold stress, the external application of GB externally enhances the ability of tomato seedlings to scavenge excess ROS by strengthening the enzymatic antioxidant defense system to varying degrees, and the effect is the most significant at a concentration of 30mmol/L.

### Exogenous application of GB enhances the photosynthetic capacity and cold tolerance of tomato seedlings

**Figure 3:**
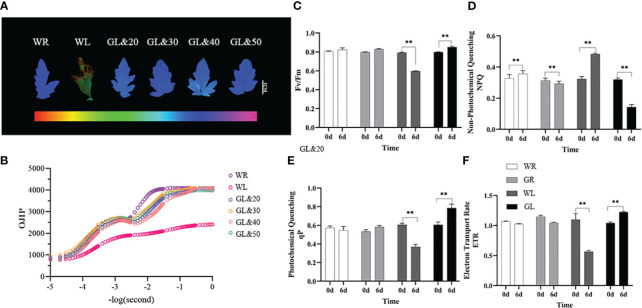
Effects of different concentrations of GB on photosynthetic capacity of tomato seedlings under cold stress **(A)** Chlorophyll fluorescence. **(B)** Chlorophyll fluorescence kinetic curve (OJIP) **(C)** Maximum photochemical efficiency of PSII (Fv/Fm). **(D)** Non-photochemical quenching coefficient (NPQ). **(E)** Photochemical quenching coefficient (qP). **(F)** Apparent photosynthetic electron transport rate (ETR). In all cases, the asterisk indicated a significant difference among the groups according to Tukey’s test. (**P < 0.01). WR: tomato seedlings treated with water alone at room temperature; WL: Tomato seedlings treated with water at low temperature; GL&20: 20mmol/L GB was applied externally at low temperature; GL&30: 30mmol/L GB was applied externally at low temperature. GL&40: 40mmol/L GB was applied externally at low temperature; GL&50: 50mmol/L GBe was applied externally at low temperature; GL: Tomato seedlings treated with 30mmol/L GB at low temperature.

The photosynthetic system was observed and analyzed by a chlorophyll fluorescence imaging system (WALZ, IMAG-MAX/L) in the same part of tomato leaves. Chlorophyll fluorescence phenotype analysis results showed that the tomato leaves treated with WL were significantly damaged, and the photosystem II (PSII) activity was disrupted. In contrast, tomato plants treated with GB (20mmol/L, 30mmol/L, 40mmol/L, and 50mmol/L) grew well, with no apparent damage (**Figure 3A**). Subsequently, the data analysis of the fluorescence yield at various points of the OJIP chlorophyll fluorescence kinetics curve of the leaves revealed that the PSII reaction center activity was reduced. Nevertheless, there was almost no difference in the leaves treated with GB at low temperature compared to the control at room temperature, and the leaves appeared a normal physiological phenotype, with the activity of light and PSII significantly improved (**Figure 3B**). It was found that the application of GB with a concentration of 30mmol/L had the most significant alleviating effect. To further investigate the effects of GB treatment, the chlorophyll fluorescence-related parameters in tomato leaves were measured using the plant efficiency analyzer before and on the sixth day of low-temperature treatment. It shows that the chlorophyll fluorescence-related parameters of the tomato leaves treated with water were all inhibited in photosynthesis. The maximum photochemical efficiency of PSII (Fv/Fm value) decreased significantly (*P* < 0.01), while that of GL treatment increased significantly compared to WL treatment, indicating that GB treatment helps prevent PSII photo-inhibition caused by cold stress (**Figure 3C**). In addition, NPQ, ETR (II), and qP increased significantly compared to WL treatment (*P* < 0.01) (**Figures 3D–F**). These results suggest that cold stress reduces the capacity of light energy absorption and the oxygen release of PSII. However, external application GB can maintain the stability of PSII under cold stress by dissipating excess light energy through NPQ, which maintains its structural integrity and reduces light damage caused by cold stress. Furthermore, GB treatment helps in improving the rate of photosynthetic electron transfer, enhancing the photochemical activity under cold stress, which protects PSII from light damage.

### Effects of exogenous GB on high-flux plant phenotypes of tomato seedlings under cold stress

**Figure 4:**
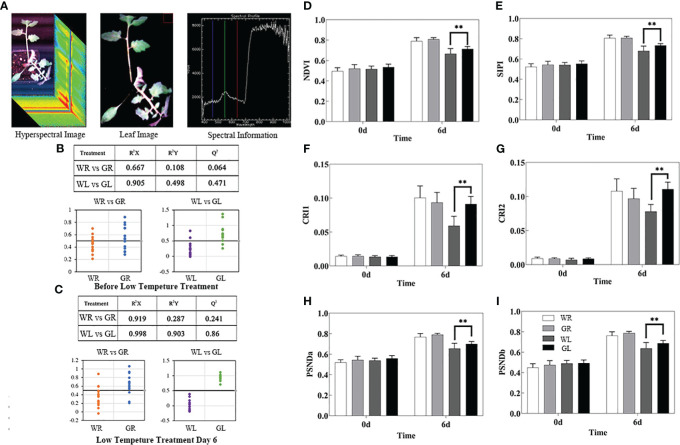
Effects of different concentrations of GB applied on high-throughput plant phenotyping of tomato seedlings under cold stress. **(A)** Acquisition and processing of hyperspectral data. **(B)** Classification results of PLS-DA model and PLS-DA model of spectral reflectance before low temperature treatment. **(C)** The spectral reflectance classification results of PLS-DA model and PLS-DA model on the 6th day of low temperature treatment (0.5 was taken as the threshold value, WR and WL categories were set as the value 0, GR and GL categories were set as the value 1, and the y-coordinate was the predicted value of the model for the category values; The closer the predicted value was to the set value, the better classification effect was and the higher distinction between the two categories). **(D)** NDVI biomass growth status. **(E)** SIPI. **(F)** CRI1. **(G)** CRI2. **(H)** PSNDa. **(I)** PSNDb. In all cases, the asterisk indicated a significant difference among the groups according to Tukey’s test (**P < 0.01). WR: tomato seedlings treated with water at room temperature; GR: tomato seedlings treated with 30mmol/L GB at room temperature; WL: Tomato seedlings treated with water at low temperature; GL: Tomato seedlings treated with 30mmol/L GB at low temperature.

A hyperspectral imaging system was used to obtain hyperspectral images of the tomato seedlings (**Figure 4A**). No significant differences were found in the spectral reflectance in the four treatments before low temperature treatment, as well as between CB treatment and the control for day 6 at normal temperature (**Figures 4B, C**). Compared with GL treatment, the spectral reflectance of WL treatment was R^2^Y=0.899 and R^2^Q=0.86, indicating the significant differences of the spectral reflectance of WL treatment and GL treatment under cold stress. The above results showed that tomato leaves were severely damaged under cold stress, and the external application of GB could significantly alleviate the damage. Additionally, the NDVI value of the low temperature treatment group was lower than that of the control group, indicating that the growth was inhibited. However, the NDVI value of GB treatment group was significantly higher (*P* < 0.01) than that of low-temperature water treatment (**Figure 4D**). Carotenoid content, as measured by SIPI, CRI1 and CRI2, showed a positive correlation with growth accumulation. However, the overall carotenoid content of low-temperature treatment group was lower than that of normal temperature group, while the GB treatment group exhibited higher carotenoid content than the low-temperature water treatment group (**Figures 4E–G**). Chlorophyll a and b content, as measured by PSNDa and PSNDb, respectively, showed significantly higher levels (*P* < 0.01) in the GB treatment group compared to low-temperature water treatment group (**Figures 4H, I**). In conclusion, it highlights the significant damage to tomato seedling leaves under cold stress, and demonstrates that the external application of GB can effectively mitigates the detrimental effects of the cold stress.

### The effect of GB on GA and ABA content in tomato seedlings

**Figure 5:**
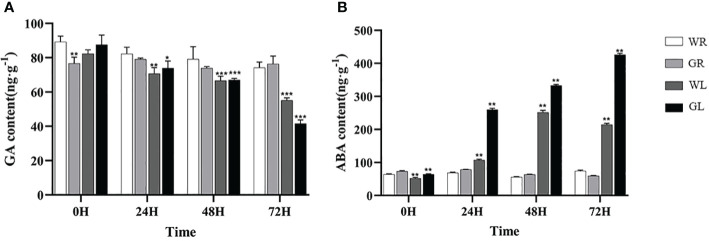
Effects of different concentrations of GB on plant hormone levels in tomato seedlings under cold stress **(A)** GA content **(B)** ABA content. In all cases the asterisk indicates a significant difference among the groups according to Tukey’s test (*P < 0.05, **P < 0.01, *** *P* < 0.001). WR: tomato seedlings treated with water alone at room temperature; GR: tomato seedlings treated with 30mmol/L GB at room temperature; WL: Tomato seedlings treated with water at low temperature; GL: Tomato seedlings treated with 30mmol/L GB at low temperature.

After cold stress, the GA contents of all plants gradually decreased. As time goes on, the content of GA in the seedlings treated with GB is the lowest (*P* < 0.01) (**Figure 5A**) ABA content gradually accumulated in tomato seedlings treated with WL and GL, and the ABA content in tomato plants treated with WL began to decrease at 48h of stress, inhibiting ABA compound synthesis. On the contrary, the ABA content in tomato seedlings treated with GL significantly increased compared to WL treatment (*P* < 0.01) (**Figure 5B**), with an increase of 138.1%, 32.4%, and 97.6% at 24, 48, and 72h of treatment, respectively, and the ABA synthesis pathway was activated, reaching its peak at 72h of cold stress.

### Effects of GB on cold-regulated genes expression in tomato seedlings

**Figure 6:**
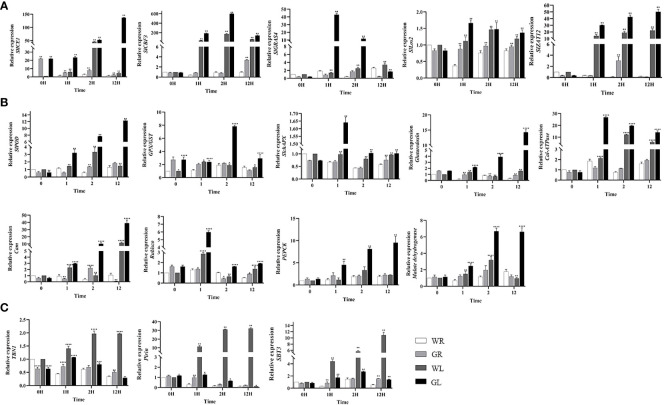
Effect of GB treatment on cold regulation genes expression in tomato seedlings under cold stress. **(A)** Cold regulatory genes *SlICE1, SlCBF3, SlGRAS4, Los2, ZAT12*. **(B)** Genes involved in antioxidant capacity (*SlPOD, GPX/GST, SlAPX,Glut*), calcium signaling pathways (*Cal-ATPase*) and calmodulin-binding protein (*Cam*), photosynthesis (*Rubsico*), energy metabolism (*PEPCK*) and *MDH*. **(C)** Genes related to programmed cell death (*Pirin*, *TBN1*, and *SBT3*). The bar is represented as the average of three repeated calculations. In all cases, an asterisk indicated a significant difference among the groups according to Tukey’s test (*P < 0.05, **P < 0.01, *** *P* < 0.001, **** *P* < 0.0001). WR: tomato seedlings treated with water alone at room temperature; GR: tomato seedlings treated with 30mmol/L GB at room temperature; WL: Tomato seedlings treated with water at low temperature; GL: Tomato seedlings treated with 30mmol/L GB at low temperature.

To further explore the molecular mechanisms of GB on the cold tolerance of tomato seedlings, we analyzed the expression levels of cold-regulated genes under cold stress. Our results showed that the expression levels of *SlGRAS4, ICE1, SlCBF3, Lox2*, and *ZAT12* in tomato plants treated with GB were significantly up-regulated (*P* < 0.01) compared to those treated with water, reaching their maximum levels after 1h, 12h, 2h, 1h, and 12h of cold stress, respectively (**Figure 6A**). However, the expression of *SlCBF1* and *SlCBF2* genes was not detected due to their low abundance. *SlGRAS4* which plays an important role in tomato cold resistance was also tested and found to be regulated. At the same time, it was also found that the gene expression level in the SlGRAS4 pathway including antioxidant capacity (*SlPOD, GPX/GST, Glut*, and *SlAPX*), calcium signaling pathways (*Cal-ATPase* and *Cam*), photosynthesis (*Rubsico*), and energy metabolism (*PEPCK* and *MDH*), has changed as shown in **Figure 6B**. Our results demonstrated that during cold stress, the expression levels of genes associated with antioxidant capacity (*SlPOD, GPX/GST, SlAPX*, and *Glut*), photosynthesis (*Rubsico*), and energy metabolism (*PEPCK* and *MDH*) in tomato plants treated with GB were significantly up-regulated compared to those treated with water (*P* < 0.01). The expression of calcium signaling pathways genes (*Cal-ATPase* and *Cam*), was notably significantly up-regulated in GL-treated plants compared to WL-treated plants (*P* < 0.01). Moreover, we measured the expression levels of *Pirin, TBN1*, and *SBT3* which associated with programmed cell death (PCD) and found that the expression levels of three PCD genes were elevated in tomato plants treated with water, reaching their highest levels after 12h of cold stress. Instead, the expression of these three PCD genes was significantly down-regulated (*P* < 0.01) in tomato plants treated with GB, showing significant differences (**Figure 6C**). Our results indicate that low temperature leads to the expression of genes related to PCD, and GB treatment can mitigate the extent of PCD under cold stress, thereby enhancing the cold tolerance of tomato seedlings.

## Discussion

Cold stress is well-known in affecting plant growth and development, resulting in suppressed seed germination, impaired growth, compromised reproductive capacity, reduced crop and yield and quality (Wang et al., 2022). Tomato shows high sensitivity to low temperature at all stages during growth. The use of exogenous agents in agricultural production can enhance cold tolerance, promote growth, and increase crop yield. The application 24-epi-brassinolide (EBR) on tomato seedling induced oxidative stress and changed the content of endogenous phytohormones under low-temperature (Heidari et al., 2021b). In our previous study, exogenous GB was found to successfully enhance the seed germination rate under cold stress (Zhang et al., 2022). Here, the appropriate exogenous GB was also found to effectively mitigate the effects of cold stress on tomato seedlings and significantly improve the survival rate.

Cold stress induces physiological and biochemical changes including photosynthesis, respiration, and ROS, including O_2_^-^ and H_2_O_2_ (Hayat et al., 2022). To counteract the detrimental effects of ROS, plants activate protective enzyme systems, including SOD, POD, and CAT to reduce oxidants and regulate cellular homeostasis (Hasanuzzaman et al., 2020; Kamran et al., 2020). In this study, the accumulation of H_2_O_2_ in the leaves appeared altered by different treatment under low temperature. The tomato seedling treated with GB had significantly lower levels of H_2_O_2_, O_2_^-^, and MDA, as while as higher level of SOD, POD, and CAT than water-treated tomato seedling under cold stress (**Figure 2**). In addition, we also analyzed the gene expression levels of *SlPOD, GPX/GST, SlAPX*, and *Glut*, which is involved in antioxidant activity regulation. Their expression was significantly up-regulated after treatment of exogenous GB under cold stress compared with the control. The accumulation of proline has also been substantiated to enhance plant resistance (Hayat et al., 2012). We also found the proline content, which was reported to reduce lipid peroxidation and acts as an antioxidant to overcome the oxidative stress created by cold stress (Heidari et al., 2021b), was also significantly increased. Collectively, it can be inferred that exogenous GB can serve as a cryoprotectant to regulate the cold tolerance of plants by activating the enzymatic antioxidant defense system to scavenge excess ROS and alleviate the inhibitory effects of chilling stress.

Plant photosynthesis as a crucial process in plants is highly susceptible to temperature. PSII is recognized as the main component inhibited by temperature stress, while chlorophyll a fluorescence transient has been widely used to study PSII performance in plants under environmental stresses (Devacht et al., 2011; Yan et al., 2013; Kalisz et al., 2016). In our study, the expression of *Rubsico*, the photosystem-related gene, was significantly up-regulated with exogenous GB treatment under cold stress The photoinhibition of PSII in tomato plants treated with exogenous GB was effectively alleviated (**Figure 3**). Fv/Fm, ETR, and qP in PSII chlorophyll fluorescence were significantly increased, while the value of NPQ was decreased, indicating reduced energy dissipation and enhanced electron transfer activity. Our findings revealed that exogenous cryoprotectants can maintain the stability of PSII under cold stress, maintaining its photochemical activity and enhancing the rate of photosynthetic electron transfer.

In recent years, plant phenomics offers a suite of new technologies to investigate crop breeding and to environmental responses (Pasala and Pandey, 2020; Wang et al., 2023). High-throughput plant phenotyping approaches are developing rapidly and are already helping to bridge the genotype phenotype gap (Hall et al., 2022). Recently, the use of an automated high-throughput phenotyping platform to analyze the dynamics of maize growth provides a valuable tool for molecular design breeding and predicting maize varieties with ideal plant architectures (Wang et al., 2023). Here, we analyzed the effect of GB on tomato seedlings under cold stress using the high-throughput phenotyping tools. Our finding revealed that GB could significantly alleviate the damage caused by low temperature and keep the normal growth and development of seedlings for image analysis. Our high-throughput phenotype data is consistent with other molecular, physiological and metabolic characteristics. Taken together, combining plant phenomics with other methods is a novel approach for dissecting plants characteristics under low temperature stress, which will be useful for studying the mechanisms of abiotic stress in crops.

Phytohormones play central roles in the ability of plants to adapt to changing environments by regulating growth, development, nutrient allocation, and source/sink transitions (Peleg and Blumwald, 2011). The ABA signaling pathway and other hormonal interactions have been identified as crucial roles in enabling plants to respond to various abiotic stresses (Peleg and Blumwald, 2011; Finkelstein, 2013; Rezaul et al., 2019). On the other hand, the balance between GA and ABA mediates plant developmental processes in conferring stress resistance (Vishal and Kumar, 2018). ABA positively regulates transcriptional control and metabolomics alterations enhanced tolerance to cold when maize encountered extreme temperatures (Guo et al., 2021). Under low-temperature conditions, DELLA proteins, as the negative transcription factor in GA pathway, are components of the CBF1-mediated cold stress response and promote plant adaptation to cold stress in rice (Achard et al., 2008). In this study, tomato seedlings treated with GB exhibited higher ABA and less GA levels compared to those in the water treatment group. Consequently, the changing of GA and ABA levels after GB treatment may ultimately enhance the survival and adaptability of tomato plants under cold stress. These results further indicated that the cross-talk between plant hormones plays a crucial role in the response of plants to abiotic stress.

Cold-induced second messengers such as Ca^2+^ signal and ROS activate the expressions of cold-responded genes. OsCNGC9, a cyclic nucleotide-gated channel, positively regulates the cold-induced calcium influx and cytoplasmic calcium elevation to enhance chilling tolerance in rice (Wang et al., 2021). In the current study, the expressions of *Cal-ATPase* and *Cam* involved in calcium signaling pathway were significantly up-regulated under cold stress. Previous studies have revealed that the CBF/DREB1-dependent transcriptional regulatory pathway is essential for plant responses to cold stress (Jaglo-Ottosen et al., 1998; Gilmour et al., 2000; Shi et al., 2018). The expression of *CBFs* rapidly induced by various transcription factors, such as *ICE1* and *GRAS4* under cold stress (Chinnusamy et al., 2003; Kim et al., 2015; Liu et al., 2020b). However, the RNA-seq results of leaf tissues under cold stress showed differences in the gene expression, alternative splicing events, and miRNA between two tomato species with different cold tolerance capacities (Chen et al., 2015). In our study, we have observed significant up-regulation of cold response pathway-related genes, including *SlCBF3*, *SlICE1*, *SlLos2*, *SlGRAS4* and *SlZAT12*, after exposure to cold stress. While, *SlCBF1* and *SlCBF2* genes showed no detectable expression levels. The above results suggested that *CBF* genes were involved in responses to cold stress, but their functions differ little in tomato and GB also probably controls COR genes through a CBF-dependent pathway in tomato in response to low-temperature stress. Both abiotic and biotic stressors can trigger programmed cell death (PCD) (Lee et al., 2014; Wang et al., 2015). PCD-related genes including *Pirin, TBN1, SBT3*, play important roles for proper growth, development and biotic/abiotic stress in plants (Orzaez et al., 2001; Koval et al., 2013; Buono et al., 2019). We observed a significant upregulation of the three PCD genes (*Pirin, TBN1, SBT3*) under WL treatment, while PCD genes were significantly down-regulated after GL treatment. In plants, PCD is a part of the defense responses against environmental stresses (Locato and De Gara, 2018). Overall, changes in the expression patterns of PCD related genes indicate that plant PCD is involved in tomato response to cold stress.

## Conclusion

**Figure 7:**
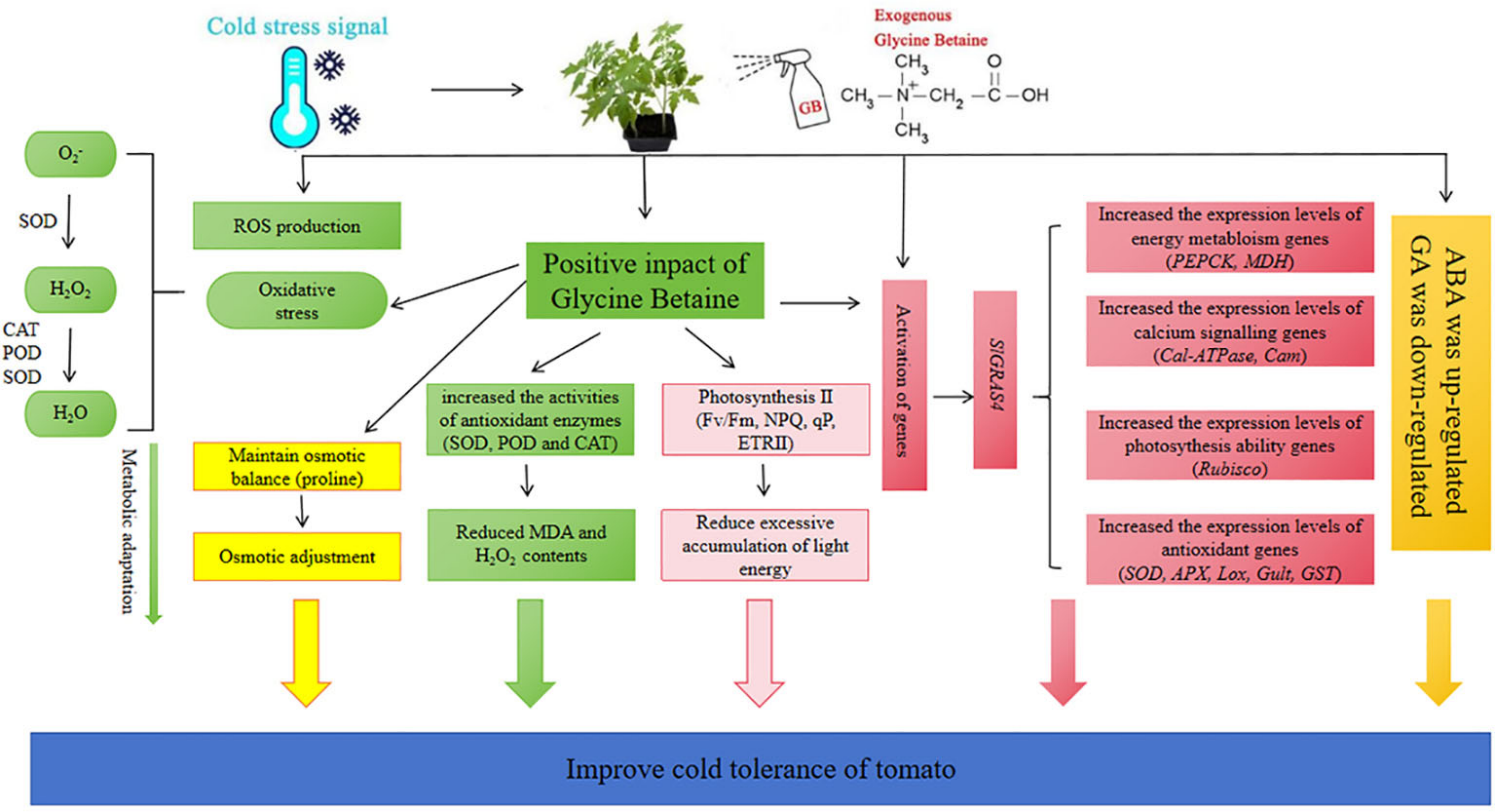
A mechanism diagram shows the mechanism of cold stress tolerance in tomato seedlings mediated by GB. Exogenous GB not only regulates antioxidant system, osmotic regulation, hormone levels, light and system II stability; It also enhances cold tolerance in tomato by regulating signal networks to activate stress-responsive genes.

Low temperature is a major limiting factor for the growth and reproduction of crop. Using certain exogenous cryoprotectants to enhance crop tolerance to temperature stress is one of the effective ways to protect plant development and agricultural production. This study investigated the effect of exogenous GB on tomato cold sensitive species under cold stress. A working model illustrated the role and complex mechanism of GB to enhance tomato tolerance under cold stress (**Figure 7**). Exogenous GB activates the expression of different genes expression. which in turn regulates its target metabolism and signaling pathways, leading to enhanced cold tolerance. The results depicted that GB regulated the expression of antioxidant system, photosynthetic system, calcium signaling, energy metabolism-related and low temperature pathway-related genes and so on in tomato plants. Moreover, GB application increased the content of antioxidant enzymes to reduce ROS and protected the photosynthetic system under cold stress. GB treatment could increase the content of proline, ABA and decrease the content of MDA, GA in response to low-temperature stress. In conclusion, GB, as a cryoprotectant, can improve the growth of tomato seedlings under cold stress through complex biological processes and multifaceted mechanism.

## Data availability statement

The original contributions presented in the study are included in the article/**Supplementary Material**. Further inquiries can be directed to the corresponding authors.

## Author contributions

TD: Writing – original draft, Writing – review & editing. SB: Writing – original draft, Writing – review & editing. LH: Writing – original draft, Writing – review & editing. LL: Writing – original draft, Writing – review & editing. YYZ: Writing – original draft, Writing – review & editing. YCZ: Writing – original draft, Writing – review & editing. WZ: Writing – original draft, Writing – review & editing.
