# Supplementary material for: Effects of exogenous glycine betaine on growth and development of tomato seedlings under cold stress
Source: Front Plant Sci. 2024 Mar 22;15:1332583. doi: 10.3389/fpls.2024.1332583 (PMC10995342; doi:10.3389/fpls.2024.1332583)
Supplement: Supplementary file 1 [file Table_1.docx]

**Supplementary Table 1** Gene accession numbers and primer sequences described in this study

| Gene Name | Forward (5’-3’) | Reverse (5’-3’) |
| --- | --- | --- |
| *LOS2* (Solyc09g009020) | GGTACCACATTTTAGTCCCTCAT | CTCGAGGATCTATGAACAGAGAA |
| *SlPOD* (Solyc02g094180) | AAAGGCAAAACAAGCAGTAGAG | ACAACATCTCTAGTGGCGATAG |
| *GPX/GST* (Solyc07g056480) | GGATATTGCGTTGATTGGGTAC | TGTCCCTCAGCATACAATTCTT |
| *SlAPX* (Solyc08g014000) | GAGTTGTTGCTGTTGAGGTAAC | GTCCCTCTTCTGGTGAACTTAA |
| *Glutaredoxin* (Solyc10g008150) | TTTAGACGACGTGGATGTTGTA | AGCTTCCATAATCCGATCCAAT |
| *Calcium-transporting ATPase* (Solyc02g064680) | AGACTGCTATATTGGAGTTCGG | GCCAAAATAATTTCTGAAGCGC |
| *Calmodulin-binding protein* (Solyc07g040710) | TATCACGAAGCCGAAGAAGAAT | GCCACAACAGAATCTTGAACTT |
| *Rubisco* (Solyc02g077860) | TGCCGAGATAATGGTCTACTTC | GCTCTAGCTAATTGTCCATCCT |
| *PEPCK* (Solyc04g076880) | TTATGTGTTATTGGTCCGGAAG | TCACCAGAAAACAAACACGATC |
| *Malate dehydrogenase* (Solyc01g090710) | TCAATGTTGCTGTTATGGTTGG | CAACAACCAAAACCTTGCAATC |
| EIF (Solyc03g005870) | ATCCTTCAGAGCGGTGTTCA | ATCTCAAGAGCCTCTGGTGG |
| *GRAS4* (Solyc01g100200) | CTCGAGACGAATTAGTTGGTGTG | GTCGACGAAATCAAAGCAATCCCA |
| *ZAT12* (Solyc06g075780) | GGTACCGATGAATGCTGACCTT | CTCGAGGTATTTGTATAATTGTTGAG |
| *SlCBF1* (Solyc03g026280) | GGTACCAGAAGTTGATGAATGCTG | CTCGAGGTATTTGTATAATTGTTGAG |
| *SlCBF2* (Solyc03g124110) | GGTACCAGCCTAATGAGCAAGA | CTCGAGGGATGGACGTTTTTGG |
| *SlCBF3* (Solyc03g026270) | GGTACCTCCCTCTTCGATTTTCAT | CTCGAGGTTATGGTAAAATGTGA |
| *SlSOD* (Solyc01g067740) | ACATACAAAAATGGTGAAGGCC | AGGATTGTAATGTGGTCCTGTT |
| *SBT3* (Solyc01g087850) | ACGAGCCTTTGTCAGGGATT | CCAGGGTTGGGAAATGTAGC |
| *Pirin* (Solyc09g098160) | GCAGTGGATGACAGCAGGAA | GTCTCGGGGATCGGTTGATG |
| *TBN1* (Solyc02g078910) | GACAAGCCCTCTACACTTCA | CACCAGCAACACACATATCC |
| ICE1 (Solyc03g118310) | ATGCTGCGATCGGTTGTCCC | GATGGAAGCTGGTTGCGGGA |
